# Tai Chi Chuan Exercise for Patients with Cardiovascular Disease

**DOI:** 10.1155/2013/983208

**Published:** 2013-11-17

**Authors:** Ching Lan, Ssu-Yuan Chen, May-Kuen Wong, Jin Shin Lai

**Affiliations:** ^1^Department of Physical Medicine and Rehabilitation, National Taiwan University Hospital and National Taiwan University, College of Medicine, 7 Chung-Shan South Road, Taipei 100, Taiwan; ^2^Department of Physical Medicine and Rehabilitation, Chang-Gung Memorial Hospital and Department of Physical Therapy, Post-Graduate Institute of Rehabilitation Science, Chang-Gung University, Taoyuan 333, Taiwan

## Abstract

Exercise training is the cornerstone of rehabilitation for patients with cardiovascular disease (CVD). Although high-intensity exercise has significant cardiovascular benefits, light-to-moderate intensity aerobic exercise also offers health benefits. With lower-intensity workouts, patients may be able to exercise for longer periods of time and increase the acceptance of exercise, particularly in unfit and elderly patients. Tai Chi Chuan (Tai Chi) is a traditional Chinese mind-body exercise. The exercise intensity of Tai Chi is light to moderate, depending on its training style, posture, and duration. Previous research has shown that Tai Chi enhances aerobic capacity, muscular strength, balance, and psychological well-being. Additionally, Tai Chi training has significant benefits for common cardiovascular risk factors, such as hypertension, diabetes mellitus, dyslipidemia, poor exercise capacity, endothelial dysfunction, and depression. Tai Chi is safe and effective in patients with acute myocardial infarction (AMI), coronary artery bypass grafting (CABG) surgery, congestive heart failure (HF), and stroke. In conclusion, Tai Chi has significant benefits to patients with cardiovascular disease, and it may be prescribed as an alternative exercise program for selected patients with CVD.

## 1. Introduction

Regular exercise is beneficial to cardiovascular health and longevity. The Centers for Disease Control and Prevention and the American College of Sports Medicine (ACSM) recommend a minimum of 30 minutes of moderate-intensity physical activity on most days of the week [1]. According to the National Institutes of Health-American Association of Retired Persons (NIH-AARP) Diet and Health Study [2], achievement of activity levels approximate the recommendations for moderate activity (at least 30 minutes on most days of the week) may decrease 27% of mortality in men and women. In developing countries, the fast increase of CVD mortality may partly be attributed to the decrease of physical activity. The death rate of CVD in China, for example, nearly increased 50% from 1990 to 2009, and the decrease of exercise participation played an important role in the increase of mortality. A recent investigation in 9 provinces and cities of China showed that the physical activity in 2006 declined by 27.8% in men and by 36.9% in women in comparison with those in 1997 [3].

Nonvigorous exercise training is a central focus of health promotion and is the core component of rehabilitation for patients with CVD. According to a recent meta-analysis evaluating the effect of light-to-moderate physical activity [4], 2.5 hour/week moderate-intensity activity (equivalent to 30 min exercise 5 days a week) compared with no physical activity was associated with a reduction in mortality risk of 19%, while 7 hour/week of moderate activity compared with no activity reduced the mortality risk by 24%. 

Tai Chi Chuan (Tai Chi) is a Chinese traditional mind-body exercise. Although the exercise intensity of Tai Chi is low to moderate [5, 6], previous studies have shown that it offers benefits for aerobic capacity [7–9], muscular strength [10, 11], balance, and cardiovascular risk factors. Further, Tai Chi appears to be safe and effective for patients with acute myocardial infarction, coronary artery bypass grafting surgery, congestive heart failure, and stroke. From the standpoint of exercise prescription, Tai Chi is a suitable exercise for patients with CVD because it is easily accessible and of low cost and can be easily implemented in the community setting. The aim of this literature review is to provide an overview of Tai Chi benefits on cardiovascular health and to introduce the potential application of Tai Chi for patients with CVD.

## 2. Effect of Tai Chi on Cardiovascular Risk Factors

### 2.1. Hypertension

Hypertension is a major risk factor of coronary artery disease, heart failure, stroke, and peripheral vascular disease. About 54% of stroke and 47% of ischaemic heart disease worldwide were attributable to hypertension [12]. Lowering blood pressure (BP) in hypertensive individuals significantly reduces cardiovascular morbidity and mortality. Regular aerobic exercise and lifestyle change are important for preventing and treating high blood pressure. Systemic review of randomized clinical trials indicated that aerobic exercise training leads to reductions in resting BP of 5–7 mm Hg [13] and the reductions appear to be more pronounced in hypertensive subjects [14, 15]. The American College of Sports Medicine recommends the following exercise guidelines for individuals with hypertension: (1) frequency: aerobic exercise on most, preferably all days of the week; resistance exercise 2-3 days per week; (2) intensity: moderate-intensity aerobic (i.e., 40%–60% heart rate reserve or oxygen uptake reserve) and resistance exercise (60–80% of one repetition maximum); (3) time: 30–60 minutes per day of aerobic exercise and resistance training at least one set of 8–12 repetitions for each of the major muscle groups [13]. 

Tai Chi is a moderate-intensity exercise program fulfilling the ACSM recommendations and thus Tai Chi may be beneficial to individuals with hypertension. In most of the Tai Chi intervention studies, 6- to 12-week training programs have been shown to lower the systolic and diastolic BP at rest or after exercise, and hypertensive patients exhibit the most favorable improvement [16–20]. The decrease of BP during submaximal exercise may lower the rate pressure product, which indicates the decrease of myocardial oxygen consumption. In a recent pooled analysis of 26 studies (11 in English, 15 in Chinese), Yeh and colleagues [21] reported positive effect of Tai Chi on BP control. In individuals with hypertension, Tai Chi training may reduce systolic BP (range: −7 to −32 mm Hg) and diastolic BP (−2.4 to −18 mm Hg). In studies for noncardiovascular populations or healthy patients, the decreases ranged from −4 to −18 mm Hg in systolic BP and from −2.3 to −7.5 mm Hg in diastolic BP.

### 2.2. Diabetes Mellitus

Type 2 diabetes mellitus (DM) is a fast growing risk factor for cardiovascular disease. Exercise is a key to lifetime management of Type 2 diabetes or impaired glucose tolerance [22–24]. The benefits of regular exercise in individuals with Type 2 DM and prediabetes include improved glucose tolerance, increased insulin sensitivity, and decreased Glycated hemoglobin (HbA_1_C). Additionally, exercise can help prevent the development of diabetes in patients with impaired glucose tolerance. In the Da Qing Diabetes Prevention Study for people with impaired glucose tolerance [25], lifestyle intervention groups (diet and exercise) had a 43% lower incidence of DM over the 20-year follow-up period. The Diabetes Prevention Program study in America [26] also found that participants who lost a modest amount of weight through dietary changes and increased physical activity reduced the incidence of DM by 58%.

Several studies have shown the benefits of Tai Chi for diabetic patients. In a pilot study for 12 patients with DM, Wang [27] reported that an 8 wk Tai Chi program could decrease blood glucose. Additionally, high- and low-affinity insulin receptor numbers and low-affinity insulin receptor binding capacity were increased. In another study, Zhang and Fu [28] randomly assigned 20 female diabetic patients to Tai Chi or control group. The exercise protocol was one-hour Tai Chi per day and 5 days a week. After 14 weeks of training, the Tai Chi group had significantly lower fasting plasma glucose and glycated serum proteins and higher fasting plasma insulin compared to the control group. The results showed that Tai Chi could be used as an exercise intervention to improve diabetic control. For obese patients with diabetes, Chen and colleagues [29] reported that a 12-week of Chen Tai Chi training induced significant improvement in body mass index, triglyceride, and high-density lipoprotein cholesterol. In addition, serum malondialdehyde (oxidative stress indicator) and C-reactive protein (inflammation indicator) decreased significantly. 

In diabetic patients complicated with peripheral neuropathy, Ahn and Song [30] recruited 59 diabetic patients with neuropathy and assigned them into a Tai Chi group or a control group. The Tai Chi group participated in an exercise program comprised 1 hour of Tai Chi twice a week for 12 weeks. After training, patients in the Tai Chi group showed improvement in glucose control, balance, neuropathic symptoms, and some dimensions of the quality of life compared to the control group. However, the dropout rate was high (34%) in this study.

A 12-week Tai Chi program for diabetic patients might obtain significant benefits in the quality of life. In a study reported by Liu and colleagues [31], 41 patients with diabetes were randomly assigned to Tai Chi (*n* = 20) or usual care group (*n* = 21). After training, the Tai Chi group revealed significant improvements in the Short Form 36-item Health Survey (SF-36) subscales of physical functioning, role physical, bodily pain, and vitality. 

A recent meta-analysis conducted by Yan and colleagues [32] pooled 4 randomized controlled trials (RCT) and 5 nonrandomized controlled trials (NRCT) to assess the effect of Tai Chi in patients with Type 2 DM. The weighted mean differences from RCT were −14.82 mg/dL (*P* = 0.40) for fasting blood glucose, −0.19% (*P* = 0.09) for HbA1c, and −0.34 units (*P* = 0.80) for homeostasis model assessment of insulin resistance (HOMA) index (indicator of insulin resistance). The weighted mean differences from NRCT were −11.22 mg/dL (*P* = 0.003) for FBG and −0.41% (*P* < 0.00001) for HbA1c and −0.60 units (*P* = 0.16). Because most Tai Chi studies have only a small group of subjects, further large-scale randomized trials are needed to clarify the potential health effect of Tai Chi for diabetic patients.

### 2.3. Dyslipidemia

Regular exercise may ameliorate the trend toward abnormal blood lipid profile. A meta-analysis of 31 randomized controlled trials with exercise training reported a significant decrease in total cholesterol, low density lipoprotein cholesterol (LDL-C), and triglyceride (TG) and an increase in high-density lipoprotein cholesterol (HDL-C) [33].

Several studies have shown beneficial effects of Tai Chi training on lipid profile. In a study reported by Tsai and colleagues [19], 88 patients with Stage I hypertension were randomly assigned to a Tai Chi or a sedentary control group. After 12 weeks of classical Yang Tai Chi training, total cholesterol, TG, and LDL-C concentration decreased by 15.2, 23.8, and 19.7 mg/dL, respectively, and HDL-C increased 4.7 mg/dL. In healthy elderly individuals, however, Thomas and colleagues [34] reported no significant change in total cholesterol, TG, LDL-C, and HDL-C after 12 months of Tai Chi training. In another study, Zhang and Fu [28] randomly assigned 20 female diabetic patients to Tai Chi or control group. After 14 weeks of training, the Tai Chi group only showed significantly lower TG compared to the control group, whereas there were no significant differences in total cholesterol, HDL-C, and LDL-C. All these studies were not specifically focused on dyslipidemic patients, and difference among results may be attributed to differences in study design, baseline lipid concentrations, training amount and intensity, changes in body composition, or the adjunctive interventions such as diet or lipid-lowering agents.

In a recent study, Lan and colleagues [35] enrolled 70 severe dyslipidemic patients to attend a one-year Yang Tai Chi training program. The inclusion criteria were as follows: (1) patients' initial level of total cholesterol > 300 mg/dL and/or TG > 500 mg/dL prior to medical treatment, (2) patients who had received lipid-lowering medication and diet therapy for at least 6 months, but the lipid profile remained abnormal (total cholesterol > 200 mg/dL or TG > 150 mg/dL). After training, the Tai Chi group showed a significant decrease of 26.3% in TG (From 224.5 ± 216.5 to 165.9 ± 147.8 mg/dL), 7.3% in total cholesterol (from 228.0 ± 41.0 to 211.4 ± 46.5 mg/dL), and 11.9% in LDL-C (from 134.3 ± 40.3 to 118.3 ± 41.3 mg/dL), whereas the HDL-C did not increase significantly. In addition, the Tai Chi group also showed a significant decrease in fasting insulin, HOMA index, and high-sensitivity C-reactive protein. Conversely, the usual care group showed no significant improvement in these cardiovascular risk factors.

### 2.4. Low Exercise Capacity

Low exercise capacity is a strong predictor of cardiac and all-cause mortality. Myers and colleagues [36] reported that the peak exercise capacity measured in metabolic equivalents (MET) was the strongest predictor of the risk of death. Each 1-MET increase in exercise capacity conferred a 12% improvement in survival. Kavanagh and colleagues [37] have reported that the risk of death for coronary patients had inverse relationship with the values of peak oxygen uptake (${\dot{\text{V}}\text{O}}_{2\text{peak}}$) during exercise testing. ${\dot{\text{V}}\text{O}}_{2\text{peak}}$ values of 15, 15–22, and >22 mL·kg^−1^·min^−1^ yielded respective multivariate adjusted hazard ratios of 1.00, 0.62, and 0.39 of cardiac death, respectively. 

Regular Tai Chi training for older individuals may improve aerobic capacity. In a cross-sectional study, Lan and colleagues [7] reported that elderly Tai Chi practitioners displayed 18-19% greater ${\dot{\text{V}}\text{O}}_{2\text{peak}}$ than their sedentary counterpart. In a 5-year follow-up study, Lan and colleagues [38] reported that regular Tai Chi training attenuated the age-related decline in ${\dot{\text{V}}\text{O}}_{2\text{peak}}$ for nearly 40% compared with the control group. Entering a Tai Chi program can also improve the aerobic capacity for sedentary elderly individuals. After one year of Tai Chi training, elderly participants showed an increase of 16.1% and 21.3% in ${\dot{\text{V}}\text{O}}_{2\text{peak}}$ in men and women, respectively [8].

A meta-analysis on 14 studies conducted by Taylor-Piliae [39] reported that Tai Chi was effective in improving aerobic capacity. Large significant effects of Tai Chi on aerobic capacity were found for subjects enrolled in the cross-sectional studies, in both genders, among adults ≧55 years old and when comparing sedentary subjects with those in Tai Chi exercise groups. In a recent meta-analysis study [40], however, the existing evidence does not indicate that Tai Chi is an effective way of increasing aerobic capacity. This study pooled 5 randomized studies and included 124 Tai Chi participants, and the training only included 5 to 15 Tai Chi movements. Most of the training protocols were 12-week “Tai Chi calisthenics,” and the exercise intensity appeared significantly lower than classical Tai Chi. Classical Tai Chi consists of 108 movements and it takes long time to learn and practice. Lan and colleagues [5] reported that the average HR during classical Yang Tai Chi practice was 58% of the heart rate reserve (HRR) and the oxygen uptake was 55% of the peak oxygen uptake. Tai Chi participants usually need 12 weeks of intensive training (with an exercise frequency 5–7 times per week) to familiarize all movements. During the familiarization phase, the exercise intensity and amount of training are inconsistent. If the goal of Tai Chi training is to increase aerobic capacity, a classical Tai Chi program for at least 6 months may be more appropriate than short-term Tai Chi-like calisthenics. 

### 2.5. Endothelial Dysfunction

Nitric oxide (NO) is an endothelium-dependent vasodilator and plays an important role in the vasodilatory response during exercise. Lack of exercise is associated with endothelial dysfunction and arthrosclerosis due to low shear stress status. Low shear stress to the vessel wall predisposes to endothelial proliferative status and may lead to the pathogenesis of arthrosclerosis. 

Regular practice of Tai Chi may enhance endothelium-dependent dilation in skin vasculature of older individuals. Wang and colleagues [41] have reported that older Tai Chi practitioners displayed a higher skin blood flow and level of plasma NO metabolite than sedentary subjects at rest and after maximal exercise. In addition, Tai Chi subjects had higher arterial blood flow and acetylcholine-induced cutaneous perfusion than the sedentary controls.

Tai Chi training also has benefits to microcirculation. Wang and colleagues [42] measured skin blood flow and vascular conductance in elderly men before and after a maximal exercise by using impedance plethysmography. Additionally, different doses of 1% acetylcholine and 1% sodium nitroprusside were iontophoretically applied to the skin of subjects' lower legs, and cutaneous microvascular perfusion responses were determined by laser Doppler measurements. In comparison with older individuals with sedentary life, older Tai Chi participants had higher lower leg arterial blood flow (LABF), LABF in response to reactive hyperemia, and lower leg venous capacity, tone, and blood flow. Additionally, the older Tai Chi group displayed similar arterial and venous hemodynamic variables to the younger sedentary group. The older Tai Chi group showed a higher ACh-induced cutaneous perfusion and a higher ratio of ACh- to SNP-induced cutaneous perfusion than those of the older sedentary group. The results showed that regular practice of Tai Chi is associated with enhanced endothelium-dependent dilation in the skin vasculature of older individuals. Moreover, Tai Chi training may delay the age-related decline of venous compliance and hyperemic arterial response. 

### 2.6. Depression

Depression and depressive symptoms are prevalent in patients with coronary heart disease. Depression in patients with AMI showed 4–6-fold increase in risk of death than in patients with no depression [43, 44]. Major depression following AMI runs a chronic course if untreated, but the prevalence significantly decreased following exercise training [45]. Depressed coronary patients who completed rehabilitation had lower mortality compared with those who did not complete training (8% versus 30%). 

Jimenez and colleagues [46] reviewed 35 Tai Chi intervention articles and reported that Tai Chi training could improve psychological function. In those studies, 9 out of 11 studies confirmed significant improvements in mood and depressive symptoms; 7 out of 8 studies showed reduction in anger and tension; 6 out of 10 studies displayed improvements in anxiety reduction.

In a recent study, Yeung and colleagues [47] randomized 39 patients with major depressive disorder into a 12 wk Tai Chi intervention or a wait-listed control group. The results showed trends toward improvement in the Tai Chi intervention group, compared with the control group, in positive treatment-response rate (24% versus 0%) and remission rate (19% versus 0%).

Taylor-Piliae and colleagues [48] have applied a 60-minute Tai Chi program (3 times per week for 12 weeks) to 39 subjects with cardiovascular risk factors. Improvement in mood state, reduction in anxiety, anger-tension, and perceived stress were found after training. Tai Chi training also benefits psychological function for patients with heart failure. In a recent study, Yeh and colleagues [49] enrolled 100 outpatients with systolic heart failure (New York Heart Association, NYHA classes I–III) and randomly assigned them to a 12-week Tai Chi exercise group or time-matched education group. After training, patients in the Tai Chi group showed greater improvements in quality of life, exercise self-efficacy, and mood than the controls. 

## 3. Application of Tai Chi in Cardiovascular Disease

Exercise is a major component of rehabilitation for patients with cardiovascular disease. The benefits of cardiac rehabilitation (CR) exercise training include exercise tolerance enhancement, amelioration of CVD risk factors, improvement of psychological well-being, and reduction of mortality [50]. Cardiac rehabilitation usually begins during hospitalization (phase I), followed by supervised outpatient program lasting 3–6 months (phase II), and continues in a lifetime maintenance stage in minimally supervised or unsupervised setting (phase III). Tai Chi can be prescribed as an alternative exercise training program for patients who need cardiac rehabilitation. In a recent cross-sectional study, Taylor-Piliae and colleagues [51] evaluated 51 patients who participated in a CR program. Twenty-three patients attended a group-based Wu Tai Chi class plus CR, while 28 subjects attended conventional CR only. Subjects attending Tai Chi plus CR showed better balance, perceived physical health, and Tai Chi self-efficacy compared to those attending CR only. The results suggest that Tai Chi can be easily implemented in the community setting or in CR facility and may offer additional exercise options for cardiac patients.

### 3.1. Coronary Artery Disease

Acute myocardial infarction is the most common cause of mortality in patients with CVD but exercise significantly reduces the mortality rate of patients with AMI. In a recent Cochrane review [52] involving 47 studies randomizing 10,794 patients with AMI to exercise-based cardiac rehabilitation or usual care, patients receiving exercise training reduced the risk for total mortality by 13%, the risk for cardiovascular mortality by 26%, and the risk for hospital admissions by 31%. Patients recovering from AMI are recommended to receive cardiac rehabilitation services; however, many patients feel inconvenient to attend CR courses. Tai Chi is easily accessible and can be practiced individually or in group settings. Channer and colleagues [16] randomly assigned 126 patients with AMI to a Tai Chi, an aerobic exercise, or a nonexercise support group. The Tai Chi group and the aerobic exercise group participated in an 8 wk training program, attended twice weekly for three weeks and then weekly for a further five weeks. The results of this study showed that Tai Chi was effective for reducing systolic and diastolic BP and was safe for patients after AMI. 

Lan and colleagues [9] applied Tai Chi in the treatment of patients after CABG and found improvement of aerobic capacity. Patients with CABG participated in a 12-month classical Yang Tai Chi program 3 times weekly as a Phase III CR program. After training, the Tai Chi group showed a significant improvement of oxygen uptake at the peak exercise and the ventilatory threshold. At the peak exercise, the Tai Chi group showed 10.3% increase in $\dot{\text{V}}{\text{O}}_{2}$ (from 26.2 ± 4.4 to 28.9 ± 5.0 mL·kg^−1^ min^−1^) and 11.8% increase in peak work rate, while the control group did not show any improvement. At the ventilatory threshold, the Tai Chi group increased 17.6% in $\dot{\text{V}}{\text{O}}_{2}$ while the control group did not display significant change. It should be noted that even a small increase in $\dot{\text{V}}{\text{O}}_{2}$ at the ventilatory threshold improves the functional level in activities of daily living.

### 3.2. Congestive Heart Failure

Congestive heart failure (HF) is characterized by the inability of the heart to deliver sufficient oxygenated blood to tissue. Exercise training improves functional capacity and symptoms in patients with HF, and the increase in exercise tolerance may be attributed to increased skeletal muscle oxidative enzymes and mitochondrial density. Previous studies have shown that low-intensity Tai Chi training has benefits to patients with HF (Table 1). In a study by Barrow and associates [53], 52 patients with HF (NYHA classes II–III) were randomly assigned to either a Tai Chi group or a standard medical care group. The Tai Chi group practiced Tai Chi twice a week for 16 weeks. After training, the Tai Chi group did not show significant increase in exercise tolerance but had improvement in symptom scores of heart failure and depression scores compared with the control group. 

Yeh and colleagues [54, 55] reported that a 12-week Tai Chi training in patients with HF revealed improvement in quality of life, sleep stability, 6-minute walking distance, and decreased serum B-type natriuretic peptide (BNP). BNP is produced by ventricular cardiomyocytes and correlates with left ventricular dysfunction. In a recent study, Yeh and colleagues [49] randomly assigned 100 patients with systolic HF to a Tai Chi group or a control group. Tai Chi participants practiced 5 basic simplified Yang Tai Chi movements twice weekly, while the control group participated in a HF education program. After 12 weeks of training, the Tai Chi group showed greater improvements in quality of life, exercise self-efficacy, and mood. For patients with HF, low-intensity exercise such as simplified Tai Chi may increase the acceptance. Interval training protocol by using selected Tai Chi movements is suitable for HF patients with very low endurance. 

Caminiti and colleagues enrolled 60 HF patients and randomly assigned them into a combined training group (CT) performing Tai Chi plus endurance training and an endurance training group (ET) [56]. After 12 weeks of training, 6-minute walking distance increased in both groups with significant between-groups differences. Systolic BP and BNP decreased in the CT group compared to the ET group. The Tai Chi group had a greater significant improvement in physical perception and peak torque of knee extensor compared to the ET group.

In patients with heart failure with preserved ejection fraction (HFPEF), Yeh and colleagues [57] randomly assigned 16 subjects to 12-week Tai Chi or aerobic exercise. Change in ${\dot{\text{V}}\text{O}}_{2\text{peak}}$ was the same between groups, but 6-minute walking distance increased more after Tai Chi training, which implied improvement in exercise endurance at submaximal workload. Both groups had improved Minnesota Living With Heart Failure scores and self-efficacy, but the Tai Chi group showed a decrease in depression scores in contrast to an increase in the aerobic exercise group. Overall, the Tai Chi group displayed similar improvement as the aerobic exercise group despite a lower training workload. 

In a recent meta-analysis study, Pan and colleagues [58] pooled data from four randomized controlled trials (*n* = 242). The results found that Tai Chi significantly improved quality of life but is not associated with significant reduction in BNP, systolic/diastolic blood pressure, improved 6-minute walking distance, or peak oxygen uptake. Further larger randomized controlled trials are needed to prove the beneficial effects of Tai Chi to HF patients.

Depressive disorders are prevalent in patients with heart failure. Patients with depression are associated with increased mortality, clinical events, and hospitalization [59]. Depression-related somatic symptoms such as fatigue and sleep disturbances may lead to physical inactivity and create a spiraling decline in physical and cardiac function. In a recent study, Redwine and colleagues [60] assigned patients with HF to a 12-week Tai Chi training group (*n* = 16) or a usual care group (*n* = 12). After training, the Tai Chi group showed a reduction in somatic symptoms of depression but not in cognitive symptoms of depression. 

### 3.3. Stroke

Stroke results in a significant decrease in quality of life, which is determined not only by the neurological deficits but also by impairment of cognitive function. In a recent meta-analysis study, Stoller and colleagues [61] reported that stroke patients benefited from exercise by improving peak oxygen uptake and walking distance. Stroke patients usually have impaired balance and motor function, thus Tai Chi exercise may have potential benefits in rehabilitation. Studies of Tai Chi in the treatment of stroke are summarized in Table 2.

Hart and colleagues [62] enrolled 18 community-dwelling stroke patients and assigned them to a Tai Chi group or a control group. The study group received Tai Chi one hour twice weekly for 12 weeks, while the control group received conventional physical therapy. After training, the Tai Chi group showed improvement in social and general functioning whereas the control group showed improvement in balance and speed of walking. The results implied that physical therapy should be served as a main treatment program for stroke patients but Tai Chi can be used as an alternative exercise program. 

Au-Yeung and colleagues [63] randomly assigned 136 stroke patients to a Tai Chi group or a control group practicing general exercises. The Tai Chi group practiced 12 short-form Tai Chi for 12 weeks. After training, the Tai Chi group showed greater excursion in the center of gravity (COG) amplitude in leaning forward, backward, and toward the affected and nonaffected sides as well as faster reaction time in moving the COG toward the nonaffected side. The result indicated that 12 weeks of Tai Chi training improved the standing balance for stroke patients. 

Wang and colleagues [64] randomly assigned 34 stroke patients to Tai Chi exercise or conventional rehabilitation in group sessions once a week for 12 weeks. After training, significant time-by-group interactions were found for sleep quality, general health score, anxiety/insomnia score, and depression score. The results implied that Tai Chi exercise is beneficial to cognitive function in stroke patents.

In a recent study, Taylor-Piliae and Coull [65] recruited 28 stroke patients to participate in a community-based Yang Tai Chi training program. Patients practiced Tai Chi exercise ≥150 minutes/week. The results showed good satisfaction, and the adherence rates were high (≥92%). There were no falls or other adverse events. Tai Chi appears to be safe and can be considered as a community-based exercise program for stroke patients.

## 4. Conclusion

Tai Chi exercise may promote cardiovascular health and can be considered as an alternative exercise program for patients with CVD. Previous studies prove that Tai Chi is safe and effective for patients with acute myocardial infarction, coronary artery bypass grafting surgery, congestive heart failure, and stroke. In addition, Tai Chi has benefits to cardiovascular risk factors, such as hypertension, diabetes, dyslipidemia, poor exercise capacity, endothelial dysfunction, and depression. However, the study design and training protocols among Tai Chi studies vary significantly, and hence the results are difficult to compare. In future research, large-scale randomized controlled trials using standardized training protocols should be performed in accordance with the guidelines of exercise prescription for patients with cardiovascular disease.

## Figures and Tables

**Table 1:** Effect of Tai Chi in patients with heart failure.

Author	Design	Patients	Intervention	Outcomes
Yeh et al. (2004) [54]	RCT (randomized controlled trial)	30 patients with HF 64 ± 13 y/o	Tai Chi group (*n* = 15): 12-week Tai Chi exercise, twice a week for 16 weeks Usual care group (*n* = 15): pharmacologic therapy, diet, and exercise counseling	Tai Chi group showed improved quality of life scores, increased 6-min walking distance, and decreased serum B-type natriuretic peptide levels compared with patients in the control group

Barrow et al. (2007) [53]	RCT	52 patients with HF 68.9 y/o (NYHA classes II-III)	Tai Chi group (*n* = 32): 16-week Tai Chi exercise, 1-hour twice weekly Medical care group (*n* = 33): no exercise	Tai Chi group had an improvement in symptom scores of heart failure and depression scores compared with those patients in the control group

Yeh et al. (2008) [55]	RCT	18 patients with HF 59 ± 14 y/o (NYHA classes I–III, mean EF: 24 ± 8%)	Tai Chi group (*n* = 8): 12-week Tai Chi exercise, 1-hour twice weekly Usual care group (*n* = 10): pharmacologic therapy and dietary and exercise counseling	ECG-based sleep spectrogram showed that Tai Chi group had a significant increase in high-frequency coupling and significant reduction in low-frequency coupling, which indicated improved sleep stability and better disease-specific quality of life

Yeh et al. (2011) [49]	RCT	100 patients with systolic HF 67 ± 11 y/o (NYHA classes I–III, mean EF: 29 ± 8%)	Tai Chi group (*n* = 50): 12-week Tai Chi exercise program Control group (*n* = 50): time-matched education	No significant changes in 6-minute walking distance and peak oxygen uptake Tai chi group had greater improvements in quality of life, exercise self efficacy, mood, and total mood disturbance

Caminiti et al. (2011) [56]	RCT	60 HF patients 73.8 ± 6 y/o, M/F 51/9,	Combined training group (CT, *n* = 30): Tai Chi + endurance training Endurance training group (ET, *n* = 30): endurance training only All patients performed 4 sessions of exercise per week for 12 weeks	6-minute walking increased in both groups Systolic BP and BNP decreased in the CT group compared to ET CT group had a greater improvement in physical perception and peak torque of knee extensor compared to ET group

Yeh et al. (2013) [57]	RCT	16 HF patients with preserved ejection fraction 66 ± 12 y/o,	Tai Chi group (*n* = 8): 12-week Tai Chi exercise program Aerobic exercise group (*n* = 8)	Change in peak oxygen uptake was similar between groups 6-minute walk distance increased more with Tai Chi Depression scores improved more with Tai Chi Both groups had improved Minnesota Living With Heart Failure scores and self-efficacy

Redwine et al. (2012) [60]	Non-RCT	28 HF patients (NYHA class II) 67.0 ± 11.9 y/o	Tai Chi group (*n* = 16): 12-week Yang Tai Chi short form 60 minutes twice per week Usual care control group (*n* = 12)	Tai Chi group reduced total depression scores and somatic/affective symptoms of depression compared to usual care patients

**Table 2:** Effect of Tai Chi in patients with stroke.

Author	Design	Patients	Intervention	Outcomes
Hart et al. (2004) [62]	RCT (randomized controlled trial)	18 men average of 27 months after the onset of the stroke	Tai Chi group (*n* = 9): Tai Chi for 1 h twice weekly Control group (*n* = 9): group exercises focusing on improvement of balance	Tai Chi group improved in general functioning and social functioning but did not exhibit changes in balance or speed of walking Control group improved in balance, speed of walking, climbing stairs, and the Up and Go Test but showed no changes in general and social functioning

Au-Yeung et al. (2009) [63]	RCT	136 men 6 months after stroke	Tai Chi group (*n* = 56): 12 weeks of short-form Tai Chi (Sun style). 1 hour of group practice weekly; 3 hours of self-practice per week Control group (*n* = 52), breathing and stretching exercises; active mobilization of muscles and joints of the limbs and trunk	Tai Chi group showed greater COG excursion amplitude in leaning forward, backward, and toward the affected and nonaffected sides as well as faster reaction time in moving the COG toward the nonaffected side The Tai Chi group demonstrated better reliance on vestibular integration for balance control

Wang et al. (2010) [64]	RCT	34 elderly patients after stroke	Tai Chi group (*n* = 17): group sessions once a week for 12 weeks Rehabilitation group (*n* = 17): conventional rehabilitation program	No significant effects of interaction between group and time in the time courses of P300 amplitudes and latencies Significant time-by-group interactions for Sleep Quality, general health total score, anxiety/insomnia score, and depression score

Taylor-Piliae and Coull (2012) [65]	RCT	28 subjects aged 69 ± 11 years 3 months after stroke	Tai Chi group (*n* = 13): Yang style 24-posture short-form Tai Chi exercise 150 min/week for 12 weeks Usual care group (*n* = 12): weekly phone calls along with written materials for participating in community-based physical activity	No falls or other adverse events The changes in balance, endurance, and quality of life scores were in favor of the Tai Chi intervention The changes in overall physical functioning, strength, and gait speed were greater for usual care subjects
